# Supplementary material for: Neuronal tuning to threat exposure remains stable in the mouse prefrontal cortex over multiple days
Source: PLoS Biol. 2024 Jan 11;22(1):e3002475. doi: 10.1371/journal.pbio.3002475 (PMC10783789; doi:10.1371/journal.pbio.3002475)
Supplement: Attachment — Submitted filename: Sylte_et_al_response_letter.pdf [file pbio.3002475.s011.pdf]

Reviewer #1: The authors of the manuscript of "Time-invariant prefrontal activity patterns during repeated exposure to intense threat" describe the neuronal activity patterns within the mPFC during an intense threat situation (Tail Suspension Task). They record Ca2+ transients before and during repeated sessions and identify common population dynamics across TS sessions. The experiments are well described and analyzed. Overall, I support publication with a few minor changes, a number of suggestions and optional items are listed below:

We thank the reviewer for their positive comments and for the constructive feedback on our work!

1) Fig 1e-h. It is informative to show the neuronal selectivity for TS not only for the population but based on the classification (TS or BS selective in Fig 1e.). For example color labels from Fig 1e, (right) onto Fig 1f. and/or running the anaylsis of Fig 1g and 1h only for the categories of Fig 1e, (right). My understanding is that results in Fig g and h include all 827 neurons that have been identified across days and not the selective neurons only. An analysis that helps to stratify individual trajectories is welcome.

To better reflect the cell classification in the results shown in Fig. 1, we followed the reviewer's suggestion and color-coded the neurons' TS selectivity score in Fig. 1f according to their baseline- or TS-selectivity as given in Fig. 1e. In addition, we quantified the proportion of neurons with selectivity on day1 that maintains their selectivity on the following days. This analysis showed that a large fraction of neurons retains their selectivity over days, supporting the correlation analysis of Fig. 1f. Finally, as suggested by the reviewer, we repeated the analyses of correlation over days (Fig. 1g) and state decoding over days (Fig. 1h) with selective neurons only, which gave comparable results to the full population. These new data are shown in g) and h) of the revised Fig. 1.

2) While not critical the term "repeatedly active neurons" is misleading and per se not precise, finding a suitable term is not trivial, but something along the lines of "repeatedly identified neuron". Identifying the same neurons across sessions is not as precise as we may wish (and is not only linked to activity), despite the authors using state of the art and trying their best to reduce false positive and false negative results it would be welcomed if the authors could show metrics/results regarding this procedure in the supplementary figures.

We agree that the term 'repeatedly active' does not fully capture the population we analyzed. A technical limitation of 1-photon imaging is that it is not possible to tell with certainty whether neurons that are detected as active on one day but not on the following day have truly turned silent or whether they have dropped below detection threshold for technical reasons (e.g., movement out of focus). The term suggested by the reviewer, 'repeatedly identified', acknowledges that fact better. We therefore changed the wording to 'repeatedly identified' throughout the manuscript.

To better document cell registration over days, we now give an example of cells detected on a single recording day along with the overlap in neurons on subsequent sessions. Moreover, we

quantified the true positive and true negative scores of the CellReg algorithm used to register neurons over sessions. These results are now shown in the new Supplementary Fig. 2.

3) In line 156 the authors state "…and that this information is specific for coping behaviors". The use of "specific" is misleading as the authors cannot show specificity beyond the behavioural states that have been tested (TS mobile/immobile & baseline).

We indeed cannot make claims beyond the comparison between baseline and TS. As a result, we changed the sentence highlighted by the reviewer to: '...that the activity of prefrontal neurons carries more information about the current movement state during TS than during baseline and that this information *does not generalize between baseline and TS movement*.' (line 164 of the revised manuscript). Moreover, we updated the concluding sentence of the same paragraph (line 182) from 'A TS-specific coding regime thus discriminates between active and passive coping styles.' to 'A TS coding regime that differs from general motion tuning during baseline…'

4) The authors if possible should link the role of existing mPFC evidence/literature regarding the PFC control of innate behaviours responses stronger in either the discussion or the introduction.

Following the reviewer's suggestions we included a more complete description of the involvement of the mPFC in controlling stereotyped learned and innate behaviours in the introduction in line 35. Specifically, we now additionally reference the known role of mPFC in the acquisition and extinction of fear memories, in the evasion of threat in the form of foot shocks and in social dominance.

5) While not necessary, I would be interested if the authors attempted, have data or are able to show changes of mPFC activity patterns prior to mobile or immobility phases of the TS (in contrast to BS). If so, the authors could potentially support a more mechanistic interpretation of the mPFC involvement in guiding the coping mechanisms.

We acknowledge that information on activity before and after behavioral transition points are indeed very informative to report. Following the suggestion, we tested for each struggle- and immobility-selective neuron whether it already elevated its activity before behavioral transition points during TS. In agreement with previously reported data obtained with electrophysiology, we found that a subset of neurons (~25-30%) increased their activity before behavioral changes are observed while the remaining neurons either elevate their activity after transitions or do not appear to be significantly modulated by transition periods. Methodologically, this was assessed from cross-correlations between the trial-averaged responses of each cell and the movement speed. The time lags were estimated as the time point of maximal and minimal correlation for struggle and immobility neurons respectively. We considered a neuron to have significant time lag if the correlation value at the time lag were above 1 standard deviation of the distribution of correlations of all analyzed neurons of a given coping type. These data suggest that a subset of cells might indeed be involved in actively driving behavioral transitions, although direct experimental evidence for this claim needs to be gathered in future. These results are shown in the revised Supplementary Fig. 4g,h and mentioned in the main text in line 173.

6) Also not necessary, and not trivial to implement, but supportive for the interpretation could be manifold representations for the BS that resemble time-, and motor activity (movement/no-movement) wise, episodes of the TS. e.g. identify BS movement trajectories and replicate the analysis. As it is not clear what are the latent causes for the current consistency within the representations.

We agree with the reviewer that it would be informative to apply our manifold analysis to the baseline recording. Although it is possible to perform dimensionality reduction and visualize the population activity during baseline, it is unfortunately not possible to implement our manifold analysis with baseline recordings. To align two manifolds with our procedure, we would have to first compute template manifolds from averaged binned movement and immobility epochs. For this averaging to work, we would have to know exactly when an epoch begins and when it ends. Since the movement during baseline is highly non-binary and variable (for example, even clear resting could contain various movements of the head), we found it hard to divide the behavior into these two binary states. For the same reason, we only analyzed movement in a continuous manner when comparing tuning to movement during TS and baseline. Just as critical, even when we tried to binarize the behavior by using various thresholds, we found it near impossible to decide when a behavior started and ended due to the continuous change in movements.

Reviewer #2: In this interesting study, Style et al used 1-photon calcium imaging in mice to probe the activity of prefrontal cells during repeated exposure to intense threat in a tail suspension (TS) paradigm. They showed a stable coding regime during repeated TS exposure in which neurons maintain comparable TS activation profiles as well as robust and specific movement tuning over time. Furthermore, they identified a low-dimensional neural manifold which was preserved across time and individuals. In all, the manuscript is well-written, and the data are presented orderly. The data can contribute to disentangling the prefrontal networks in controlling coping styles during threat.

We thank the reviewer for their positive appraisal of our work.

I have the following suggestions and recommendations:

1) The "habituation" was defined as "a form of simple, nonassociative learning in which the magnitude of the response to a specific stimulus decreases with repeated exposure to that stimulus"[1,2]. This is an important biological question to be addressed. The authors showed behavior of mice changed during days towards longer immobility duration in the repeated tail suspension exposure, which provides a great way to study "habituation to repeated stress". The claim "Rather, immobility-preferring neurons activated more often" that in Line 294 of the discussion is exciting. But these are nowhere to be found in the results section. The related data and clear interpretation should be provided in the manuscript.

We originally chose the formulation “Rather, immobility-preferring neurons activated more often” since we did not observe major changes in the movement correlation over days (suggesting that

the cells maintain their individual struggling or immobility preference over time) while simultaneously observing longer immobility times over days. This jointly suggested to us that, considering the entire TS duration, immobility neurons would be active more often. However, we now think that this statement could be misinterpreted as the immobility neurons increasing their firing rate during immobility, which we did not quantitatively assess in our original manuscript.

To further clarify this issue and to follow the reviewer's suggestion to better analyze the habituation aspect in our data, we quantified to additional metrics: First, from the set of repeatedly identified neurons, we measured the proportion of struggling- and immobility-selective neurons over days to get a more direct estimate of the number of neurons showing a struggling-selective or immobility-selective response. In line with the large correlation in TS movement score over days, those proportions remained relatively stable, although we detected a mild and significant increase in the number of struggle cells over days. Second, we directly measured the mean calcium activity of both functionally defined populations (i.e., mean activity of struggle neurons during struggling and mean activity of immobility cells during immobility). This analysis revealed an increase in activity for struggle neurons but a decrease for immobility cells over days. Thus, while the magnitude of speed tuning remains largely constant over time, the activity levels of the different neuron groups are subject to some level of change. These new results are now shown in Supplementary Fig. 5a,b and reported in the main text in line 216. As a consequence of these new results, we removed the misleading statement mentioned above from the main text. Furthermore, we acknowledge in the discussion in line 316 that the behavioural response follows a classical habituation scheme.

2) It is appreciated that the authors revealed histological verification of lens locations in mPFC of individual mouse. It would be helpful to provide a zoomed in view to show the GCaMP6f-labeled neurons in the layer V of mPFC. In addition, the accuracy of Thy1-GCaMP6f mouse to correctly label pyramidal neurons in mPFC should be demonstrated.

In the revised Supplementary Fig. 1, we provide the requested zoomed-in view of the GCaMP6f-positive neurons. In agreement with the original publication on the mouse line used in this study (Dana et al., 2014), we find the majority of GCaMP6f-positive somata to reside in layer 5, with a smaller proportion of neurons in superficial layers. This is complemented by a more detailed analysis of the imaged neurons' location in the prelimbic, cingulate and M2 areas, as requested by reviewer 3 (their point #11).

To further support that GCaMP correctly labels pyramidal neurons in this mouse line, we performed additional analyses and experiments: First, we provide qualitative evidence by showing high-magnification images of GCaMP6f-positive dendrites possessing a large number of dendritic spines, a hallmark of pyramidal neurons. Second, we provide an illustration of reconstructed dendrites of GCamP-positive neurons, resembling a long apical dendrite extending towards layer 1 as typically observed in pyramidal cells. Finally, to get quantitative confirmation, we performed new immunohistochemical experiments to assess the co-expression of GCamP6f with Emx1 (1 mouse), a pyramidal cell marker (Chan et al., 2001), and with parvalbumin and somatostatin-positive GABAergic interneurons (3 mice). These two interneuron

populations were chosen since they represent a large fraction of local GABAergic cells. Using the CellPose algorithm (Pachitariu and Stringer, 2022), we identified GCamP6f-positive neurons and cells that express either Emx1, PV or SOM in 3-dimensional confocal image stacks of the prelimbic and cingulate cortex. Co-expression was then determined by finding cells that share at least 10% in spatial overlap between the GCamP6f and one of the marker channels. We found that among GCamP6f-positive neurons, ~88% expressed Emx1 (n=708 neurons tested from 1 mouse) while co-expression of PV (0.05% ± 0.05%, n=2412 neurons tested from 3 mice) or SOM (0.24% ± 0.18%, n=2787 neurons tested from 3 mice) was very rare. Conversely, a sizable fraction of Emx1-positive neurons was identified as GCaMP6f-positive (35.7%, n=1869 neurons tested) while few PV-positive (0.78% ± 0.40%, n=412 neurons tested) or SOM-positive neurons (2.99% ± 0.91%, n= 377 neurons tested) expressed GCaMP6f. Taken together, these new data suggest that GCaMP6f expression in our mouse line is largely restricted to pyramidal neurons. These new results are now shown in Supplementary Fig. 1.

3) In the Fig.1a, the authors showed that mice were exposed to sequences of homecage and tail suspension on days 1,2,3 and 9 with imaging on days 1,3 and 9. Why the tail suspension was performed on days 2 without imaging. Does the tail suspension on day 2 influence the performances of mice on the following days?

We originally performed both behavior and recordings on days 1, 2, 3, and 9 with the plan to assess prefrontal representations over the entire experimental timeline. The rationale for this approach was that we wanted to compare adjacent recording days with pairs of days that are separated by more time in between. However, we experienced technical problems with some animals on individual recording days (e.g., field-of-view shift), which prevented us from getting analyzable data from all mice on all days. This left us with different combinations of recorded days for each mouse. For the sake of simplicity of data presentation, we decided to include recordings from a consistent set of animals for all analysis. We therefore selected a subset that 1) contained usable recordings from the first day of TS exposure and 2) maximized the number of mice and the covered days, which resulted in the 6 mice with imaging on day 1, 3 & 9 in the final manuscript. In the revised methods section in line 388, we now provide a clear explanation for the exclusion of day 2 data.

To test whether this simplification affected the results, we explicitly compared key parameters of the representational stability, namely correlation in TS selectivity (related to Fig. 1) and TS movement scores over days (related to Fig. 3), for the day pairings day 1 vs. 2, day 1 vs. 3 and day 2 vs. 3. This new analysis was performed with a subset of mice for which imaging was successfully performed on all 4 behavioral days (n=5 animals) using all neurons identified on all recording days (n=700). We found similar correlations in TS selectivity (d1 vs. 2: $\beta = 0.77 \pm 0.08$, $p<0.001$, d1 vs. 3: $\beta = 0.83 \pm 0.07$, $p<0.001$, d2 vs. 3: $\beta = 0.76 \pm 0.03$, $p<0.001$) and in TS movement scores (d1 vs. 2: $\beta = 0.72 \pm 0.05$, $p<0.001$, d1 vs. 3: $\beta = 0.78 \pm 0.03$, $p<0.001$, d2 vs. 3: $\beta = 0.63 \pm 0.04$, $p<0.001$, linear mixed effect models, please refer to **Figure for reviewers 1** below).

Since all animals performed the sequence of TS exposures on day 1, 2, 3 and 9, we cannot directly address the reviewer's question whether TS exposure on day 2 affected the behavior. However, we think it is unlikely. In our analyzed set, immobility during TS increased sharply between days 1 and 3 while it remained relatively constant between day 3 and 9. The representational stability that we observed was, however, similar between day 1 and 3 compared to day 1 and 9, arguing against a major contribution of the changing behavior to the representation.

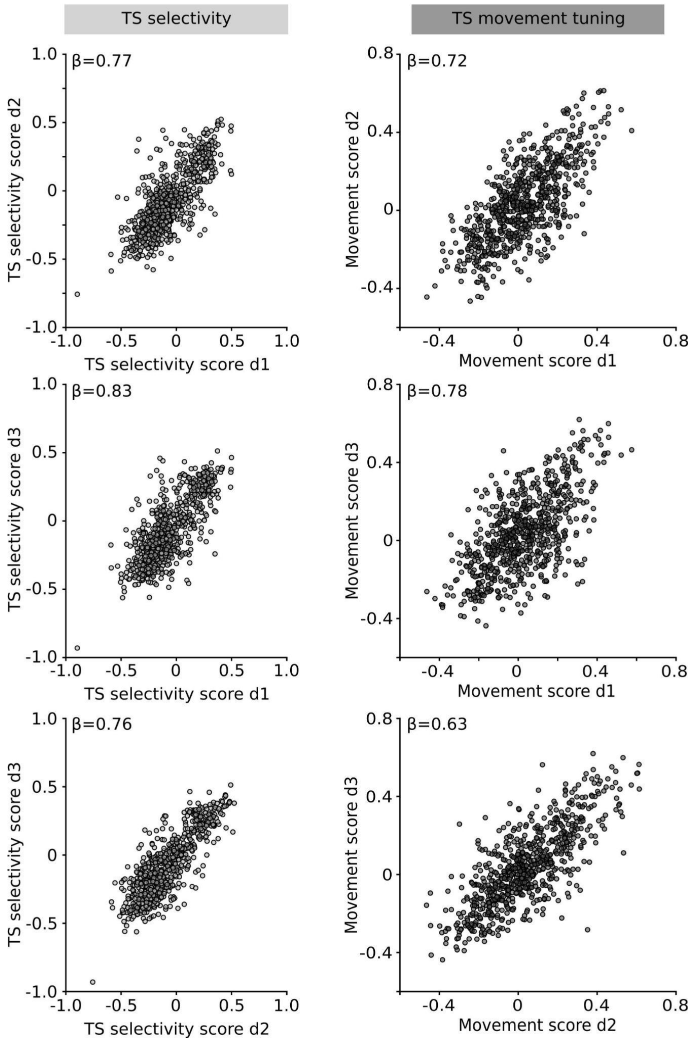

Figure for reviewers 1: Stable across-day TS selectivity and TS movement score correlations for different pairs of analyzed days.

Left: Similar correlation in TS selectivity between day 1 and 2 (top), day 1 and 3 (middle) and day 2 and 3 (bottom). Right: Correlation in TS movement scores for the same day pairings. Data are from n=700 neurons from 5 mice.

4) The obtained results were rarely analyzed by sex even when both male and female mice were used in the experiments. Sex differences in the coping styles have been reported by numerous literatures. Is there any difference in mPFC coding regime during repeated TS

exposure between male and female mice. This is an aspect the authors have to discuss in more detail.

Unfortunately, we only recorded from 2 females in this study and thus do not have the data to make quantitative comparisons on a mouse-by-mouse basis. However, we separately re-analyzed those measures which are obtained on the basis of individual neurons from females and males. These include the correlation between TS selectivity scores across days (related to Fig. 1f), correlation between baseline and TS movement score (related to Fig. 2b), and correlation of TS movement score over days (related to Fig. 3b). We observed comparable results for female and male mice, which are now reported in Supplementary Figs. 3 & 5 and mentioned in the main text in line 92. Thus, in the limits of our small sample size of female mice, our data suggest that the overall coding regime and behavioral responses are similar across sexes. We moreover indicate in the revised Fig.1c which behavioral data were obtained with males and females.

References

[1] R F Thompson, W A Spencer. Habituation: a model phenomenon for the study of neuronal substrates of behavior. Psychol Rev. 1966 Jan;73(1):16-43.
[2] Nicola Grissom, Seema Bhatnagar. Habituation to repeated stress: get used to it. Neurobiol Learn Mem. 2009 Sep;92(2):215-24.

Reviewer #3: This is an interesting manuscript analyzing the neural population dynamics during a behavior where animals are suspended and then they transition between struggling and immobility behaviors. Recordings are then done in layer 5 of the mPFC using Thy1 mice. Neurons are reportedly tracked over days. Population dynamics are then mapped onto a low dimensional manifold using isomap. Notably, the transitions between the two behavioral states can be detected in population quite stably over time. Strikingly, the transition manifold can be realigned across animals into some invariant pattering. However, the manuscript as written raises a number of questions and concerns, especially the lack of explanation about temporally conserved prefrontal activity patterns based on biological individual unit level. Overall, the results are interesting and should be of great interest to multiple fields.

We thank the reviewer for their critical and insightful comments on our work, which helped us to improve our manuscript.

1) Movement speed is quite fast relative to calcium transients, would be more persuasive to show examples and more detailed analysis of temporal alignment between calcium signals and motion speed changes.

To get more information about the temporal relationship between changes in Ca-activity and movement, we tested whether struggle- and immobility-selective neurons increased their activity level before or after behavioral transitions from immobility to struggling and from struggling to

immobility, respectively. Similar to previous reports with electrophysiology, we found that a subset of neurons (~25-30%) increases their activity before behavioral changes are observed. The remaining neurons either elevated their activity after transition points or were not significantly modulated by transition points. This was assessed from cross-correlations between the trial-averaged calcium signal of each cell and movement speed. The time lag was estimated as the time point of maximal and minimal correlation for struggle and immobility neurons respectively. We considered a neuron to have significant time lag if the correlation value at the time lag were above 1 standard deviation of the distribution of correlations of all analyzed neurons of a given coping type. These data suggest that a subset of cells might be involved in actively driving behavioral transitions, warranting further experiments to test their causal influence on behavior in future. The new results are shown in the revised Supplementary Fig. 4g,h and mentioned in the main text in line 173.

2) Could the author explain why they only focus on imaging the deep layer 5 neurons?

We focussed on deep layer neurons for technical reasons. The transgenic mouse line (Thy1-GCamP6f) used in this study shows predominant labeling in layer 5 (Dana et al., 2014). This is also apparent in the zoomed-in view of the GCamP6f-expression that is now shown in Supplementary Fig. 1 as per request of reviewer 2 (their point #2). The rationale for choosing this line was that the signal quality is excellent and, in our hands, better than upon viral transfection of pyramidal cells. That said, a direct comparison between superficial and deep layers would be highly interesting, and we think this should be investigated in future efforts.

3) The rationale for the behavioral testing and imaging timeline (Day1, 3 and 9) is not explained, especially the author compared the TS paradigm with the contextual fear conditioning paradigm (D1, D2 for short-term while one/two weeks later for long-term) in the discussion session. Why did the author perform the TS-task from Day1 to Day3 but not record on Day2?

We originally performed both behavior and recordings on days 1, 2, 3, and 9 with the plan to assess prefrontal representations over the entire experimental timeline. The rationale for this approach was that we wanted to compare adjacent recording days with pairs of days that are separated by more time in between. However, due to technical issues with some animals on individual recording days (e.g., field-of-view shift), we could not get analyzable data from all mice on all days. This left us with different combinations of recorded days for each mouse. For the sake of simplicity of data presentation, we decided to include recordings from a consistent set of animals for all analysis. We therefore selected a subset that 1) contained recordings from the first day of TS exposure and 2) maximized the number of mice and the covered days, which resulted in the 6 mice with imaging on day 1, 3 & 9 in the final manuscript. In the revised methods section in line 388, we now provide a clear explanation for the exclusion of day2 data.

To test whether this simplification affected the results, we explicitly compared key parameters of the representational stability, namely correlation in TS selectivity and TS movement scores over days, for the day pairings day 1 vs. 2, day 1 vs. 3 and day 2 vs. 3. This new analysis was performed with a subset of mice for which imaging was successfully

performed on all 4 behavioral days (n=5 animals) using all neurons identified on all recording days (n=700). We found a similar correlation in TS selectivity (d1 vs. 2: $\beta = 0.77 \pm 0.08$, $p<0.001$, d1 vs. 3: $\beta = 0.83 \pm 0.07$, $p<0.001$, d2 vs. 3: $\beta = 0.76 \pm 0.03$, $p<0.001$) and in TS movement scores (d1 vs. 2: $\beta = 0.72 \pm 0.05$, $p<0.001$, d1 vs. 3: $\beta = 0.78 \pm 0.03$, $p<0.001$, d2 vs. 3: $\beta = 0.63 \pm 0.04$, $p<0.001$, linear mixed effect models, **Figure for reviewers 1**).

4) The TS selectivity seems to be based solely on the average amplitudes of calcium signals rather than detection or counts of calcium transients based on individual neuron level. Couldn't this just detect variation due to the noise caused by movement artifacts or mis-detection of signal via CaImAn? Could the author show neural activity difference of example neurons during baseline or TS sessions? If possible, could the author also count or calculate the rate of calcium transient/event during both sessions to show the selectivity?

We thank the reviewer for pointing out this important issue. To address this point, we additionally calculated the TS preference index based on significant calcium transients rather than ‘raw’ signals. This analysis gave similar results to our original approach using the total calcium signal, with a larger proportion of neurons showing baseline than TS preference. This new result is now plotted in Fig. 1e and mentioned in the main text in line 92. In addition, we now show examples of baseline- and TS-selective neurons during baseline and TS the revised Fig. 1e.

5) While the overall stability of population dynamics is interesting, it is not clear if signal neurons are stable across trials and days? Can the authors detect single neuron responses/transients and show if single neurons maintain responses during and across days?

In the original manuscript, we provided an analysis of TS movement scores (i.e., the correlation between the cell’s calcium signal and the animal’s speed trace) of individual neurons over day 1, 3 and 9 (Fig. 3b), which suggests large stability of the movement tuning of individual neurons over days. To better reflect that aspect in the revised manuscript, we included the calcium activity alongside behavioral periods of movement and immobility during the TS of example neurons (now shown in the new panel Fig. 3a). Following the reviewers suggestion about assessing trial-by-trial stability, we additionally computed the correlation in movement score between the first and second half of the TS within day 1. Consistent with our observation across days, that analysis revealed a large correlation between the first and second half of the session ($ß=0.61 \pm 0.06$, $p<0.001$, linear mixed effects model). These data are now shown in the new Supplementary Fig. 5c and mentioned in line 223. Finally, we assessed the proportion of neurons showing a struggle- or immobility-selective phenotype (i.e., TS movement score >0.2 and <-0.2, respectively). The proportion of selective neurons increased mildly in case of struggle-responsive cells and remained unaltered over days in case of immobility-selective neurons. These new results are mentioned in line 217 and are shown in Supplementary Fig. 5a of the revised manuscript.

6) Alignment of trials is usually important for estimating low dim manifolds? How was this done?

In this study, we wanted to visualize and analyze the low dimensional population activity for each timepoint recorded. We therefore estimated the low dimensional manifold by embedding the full dataset of each TS recording session using various dimensionality reduction methods. For visualization, we color coded the manifolds to see the relative beginning and ending of each trial (struggle and immobility epochs). For each trial, we normalized an array of the length (0,1,2, … n frames) between 0 and 1 with a min-max scaler. These normalized traces were concatenated to match the calcium traces and used as color code for the plot. We admit this was insufficiently described and have therefore improved our description in the method section in line 565.

7) Registration of cells across time with non-sparse recording is challenging. Examples of tracked population templates and single unit activity patterns across sessions with spatial-temporal information would help.

Following the reviewer's proposal we included population templates showing the spatial location in the field of view of neurons detected on individual days and of those co-registered over multiple days in the new Supplementary Fig. 2. Moreover, we included an example of the spatial position of functionally identified struggle-selective neurons along with their behavior-dependent activity on each recording day. In the same figure, we now report metrics related to the cell registration (true positive and true negative scores). Finally, we provide examples showing stable struggle- and immobility-related responses of the same neurons over days in the revised Fig. 3a.

8) For the speed calculation and related decoding, since the behavioral speed was averaged between all body parts. Are there big differences between speed of different body parts? If so, will this difference also affect the decoding efficiency?

We did indeed observe differences in the movement speed of individual body parts. For example, during baseline we observed larger average speeds of 'nose' compared to 'tailbase'. Similarly, during TS, the parts 'left foot' and 'right foot' moved on average faster than 'neck' or 'camera'. The mean speed values of all individually tracked body parts are now shown in Supplementary Fig. 4i. Despite different speed values, we observed a high correlation between the speed of individual body parts, as shown in Supplementary Fig. 4i. To directly confirm that the choice of body part did not affect the prediction of movement speed during baseline or TS, we repeated our analysis predicting movement speed based on calcium signals with isolated body parts. For all cases, we observed significant decoding versus shuffled controls, comparable to our decoding from the mean speed of all body parts reported in Fig. 2f. These new data are also shown in Supplementary Fig. 4i and mentioned in line 177 of the revised main text.

9) Figure 1f: Linear mixed effects modelling is required for this. This will identify contributions and effects per animal.

We thank the reviewer for pointing out a linear mixed effect model (LME) as a more suitable statistical approach for these analyses. We reanalysed all data in which neurons were pooled across animals using LMEs (Fig. 1d,f, Fig. 2b, and Fig. 3b). For all analysis, the effects which we originally reported pooled over all mice could be reproduced with the new analysis. A description of the LME can be found in the data analysis and statistics part of the method section (line 627).

10) Figure 2, 3 and 4: All decoding is based on analysis of neural population activity, could the author perform single unit analysis or offer examples of individual unit activity, such as plot of co-firing patterns across neurons in response to changes in behavioral context/coping styles/behavioral speed, or stable tracking of sample neurons activity across days.

To better reflect the responses of individual neurons in relation to behavioral context and coping style, we provide additional analyses and visualization. First, we now show examples of calcium traces of neurons with preferential activity during baseline and TS in Fig. 1e. Second, we included a quantification of the proportion of baseline- and TS-selective neurons maintaining their baseline/TS preference over days in Fig. 1f. Third, to give a visual impression of the consistent responses of single neurons during TS, we show in the revised Fig. 2a all neurons that were simultaneously recorded in one animal on the first day of TS exposure (n=301 cells) during four subsequent epochs of struggling and immobility. The consistency in the 'trial-by-trial' responses are moreover quantified in our new analysis of within-day stability of TS movement scores (Supplementary Fig. 5c, related to the reviewer's point #5). Finally, to better emphasize the stability of individual neurons' responses over time, we included examples of the calcium signals of struggling- and immobility-selective neurons over days in the revised Fig. 3a. Furthermore, additional examples of TS responses are included in the new Supplementary Fig. 2 along with an illustration of the stable spatial footprint of the neurons in the field of view over days.

Minor Issue:
11) ExFig 1 & lens implantation: Better anatomical characterization of the brain regions and the lens implantation position are suggested. According to the implantation coordinates and lesion figure offered, with the reference from Allen Institute Adult Mouse Brain Atlas, it seems that the grin lens was located slightly shallower than author's expectation, which seems to be between the edge of M2/premotor cortex and Cg1/cingulate cortex rather than PrL/prelimbic cortex. Besides, for the diameter of the grin lens, it is possible that the imaging neurons also contains part of neurons from M2, which might also contain or encode motion/movement creatures as well as innate escaping/surviving behaviors.

We indeed observed regularly that the reached implantation depth was to some extent more dorsal than what we would expect from stereotaxic coordinates. To better describe the actual areas of the mPFC where imaging was performed, we calculated for each mouse the imaging plane based on the known focal distance of the lens and the cropping of the field of view before the extraction of calcium signals. These imaging planes are now indicated in Supplementary Fig. 1a. We then estimated for each imaged neuron whether it was located in the prelimbic

(PrL), cingulate (Cg) or M2 region. This analysis revealed that for two animals (#1 & #2), all recorded neurons stemmed from the PrL. For the other four mice, 72, 88, 74 and 80% of the imaged neurons were prelimbic in origin and 28, 12, 20 and 20% resided in Cg. A total of only 8 neurons (6%) in a single mouse (#5) were located in M2. The proportions of neurons as a function of brain area are now shown in Supplementary Fig. 1. Based on this analysis, we think that a major contribution from purely motion-encoding neurons is unlikely. Nevertheless, we quantitatively compared the behavior of neurons from the prelimbic and cingulate regions with regard to TS selectivity, the stability of their TS selectivity (related to Fig. 1e) and the stability of their TS movement score (related to Fig. 2b). These analyses revealed comparable results for both prefrontal regions, except for TS selectivity, in which Cg neurons showed slightly but significantly lower values than PrL cells. The new results are now shown in Supplementary Figs. 2 & 3, respectively, and mentioned in lines 92 and 225.

12) Fig 1g, h: It is difficult to see the individual data points and changes across days/comparison groups since the plots are so small.

We enlarged the graphs in Fig. 1g&h for better visibility.

References:

Chan C-H, Godinho LN, Thomaidou D, Tan S-S, Gulisano M, Parnavelas JG (2001) Emx1 is a Marker for Pyramidal Neurons of the Cerebral Cortex. Cereb Cortex 11:1191–1198.

Dana H, Chen T-W, Hu A, Shields BC, Guo C, Looger LL, Kim DS, Svoboda K (2014) Thy1-GCaMP6 transgenic mice for neuronal population imaging in vivo. PloS One 9:e108697.

Pachitariu M, Stringer C (2022) Cellpose 2.0: how to train your own model. Nat Methods 19:1634–1641.
